# Differential Binding of Lef1 and Msx1/2 Transcription Factors to *Dkk1* CNEs Correlates with Reporter Gene Expression *In Vivo*

**DOI:** 10.1371/journal.pone.0115442

**Published:** 2014-12-29

**Authors:** Oliver Lieven, Julia Dronka, Stephan Burmühl, Ulrich Rüther

**Affiliations:** 1 The Danish Stem Cell Center, University of Copenhagen, Blegdamsvej 3B, Building 6, 4th floor, DK-2200, Copenhagen N, Denmark; 2 Institute for Animal Developmental and Molecular Biology, Heinrich-Heine-University, Universitätsstr. 1, Building 26.13.00, D-40225, Düsseldorf, Germany; Simon Fraser University, Canada

## Abstract

Besides the active Wnt signalling itself, the extracellular inhibition by Dkk1 is important for various embryonic developmental processes, such as optic vesicle differentiation and facial outgrowth. Although a feedback crosstalk of the active Wnt/β-catenin signaling and *Dkk1* regulation has been suggested, the control of *Dkk1* transcription by the Tcf/Lef1 mediated Wnt signalling and its connection to additional signalling factors has not been elucidated *in vivo*. Here, we used a combination of transgenic mouse approaches and biochemical analyses to unravel the direct *Dkk1* transcriptional regulation via Tcf/Lefs. By using site directed mutagenesis, we tested several conserved Tcf/Lef1 binding sites within *Dkk1* conserved non-coding elements (CNEs) and found that these are required for tissue specific reporter expression. In addition a conserved Msx1/2 binding site is required for retinal reporter expression and Msx2 but not Msx1 binds its conserved binding site within CNE195 in the optic cups. Within craniofacial expression domains, Lef1 interferes with *Dkk1* directly via two conserved Tcf/Lef1 binding sites in the craniofacial enhancer CNE114, both of which are required for the general craniofacial *Dkk1* reporter activation. Furthermore, these Tcf/Lef1 sites are commonly bound in the whisker hair bud mesenchyme but specifically Tcf/Lef1 (no. 2) is required for mandibular activation and repression of maxillar *Dkk1* activation. Lastly, we tested the Tcf/Lef1 binding capacities of the *Dkk1* promoter and found that although Lef1 binds the *Dkk1* promoter, these sites are not sufficient for tissue specific *Dkk1* activation. Together, we here present the importance of conserved Tcf/Lef1 and Msx1/2 sites that are required for differential *Dkk1* transcriptional reporter activation *in vivo*. This requirement directly correlates with Lef1 and Msx1/2 interaction with these genomic loci.

## Introduction

During embryonic development, various processes, such as head induction or limb outgrowth are driven by a defined modulation of the active Wnt signalling via the extracellular inhibitor Dkk1 [1]–[5]. In addition to the relevance of the Wnt-Dkk1 crosstalk during early head induction processes, both, *Wnt* genes and *Dkk1* are dynamically expressed in various head derivates, such as the retina [6]–[8] and meso- or ectodermal domains of the 1^st^ branchial arch or craniofacial tissues [9]–[14]. These partially overlapping expression patterns strongly suggest a regulatory feedback correlation between the active Wnt signalling and *Dkk1* expression. Indeed, several studies have indicated that e.g. the outgrowth of craniofacial derivates or hair follicle formation involves a Wnt-Dkk1 crosstalk [15], [16]. These data also indicate that Wnt and Dkk1 protein levels have to be regulated in a well defined and dynamic fashion to maintain a normal tissue homeostasis. This idea is supported by the fact that a Dkk1 level reduction during craniofacial and optic cup development causes dysgenesis in these tissues, correlating with changes in Wnt-signalling activity [17]–[19]. Vice versa, the overexpression of *Dkk1* impacts on head development via Wnt signalling repression [3]. However, whether the active Wnt signalling activity is directly linked to the transcriptional regulation of *Dkk1* within head derivates has not been addressed *in vivo*. Besides the relevance for Wnt/beta catenin signalling during embryonic development, alterations of the normal Wnt or Dkk1 doses have been associated with various diseases, such as cancer and neurodegenerative disorders (reviewed in [20]). Thus, unravelling a potential Wnt-Dkk1 feedback mechanism *in vivo* would in addition improve the knowledge about *Dkk1* associated disease formation.

Canonically, extracellular binding of Wnt proteins causes a signalling transduction via two different receptors, Frizzled and Lipoprotein-receptor-related protein 5 and 6 (Lrp5/6). Thereby, Gsk3β is inactivated, causing a stabilization of β-catenin. As a consequence, β-catenin enters the nucleus and interferes with Tcf/Lef1 trancription factors [21], [22]. Tcf/Lef1 factors bind to their desired binding site within the minor groove of the DNA, causing a conformational change of the target gene regulatory sequence, resulting in a transcriptional modification of target gene expression [23]. However, the functional relevance of the Lef1 mediated DNA bending is not understood, Lef1 binding to its desired target gene sequences requires interaction with β-catenin [24] and is specified by additional transcription factors such as Smad2 and FoxH1, which bind simultaneously to their adjacent target binding sites [25].

Extracellular blocking of the canonical Wnt signaling by the secreted and soluble cystein rich protein Dkk1 via the formation of a ternary complex with Lrps and Kremen [26], [27] causes a rapid endocytosis of the Lrp receptor from the plasma membrane [27]. As a consequence of this, cytoplasmatic β-catenin is phoshorylated, recognized by Apc, Gsk3β and Axin and degraded by the proteasome. Although Dkk1 functions as a potent extracellular inhibitor of the canonical Wnt signalling, several studies revealed that the *Dkk1* gene itself is targeted by the canonical Wnt signalling via Lef1 *in vitro* [28]–[30]. These data suggest that in addition to the extracellular Wnt inhibition via Dkk1, the active Wnt signalling limits its expression level by *Dkk1* activation, promoting a negative feedback loop. Furthermore, the Lef1 mediated Wnt signaling is involved in the regulation of the homeobox transcription factor *Msx2* by direct binding to the *Msx2* promoter [31]. Since Msx1/2 transcription factors are expressed similar to Lef1 in craniofacial and optic cup expression domains (own observation), these factors might be involved in Lef1 target gene coregulation. Supporting this idea, the disruption of Msx1 and Msx2 in the mouse embryo results in hypoplasia of the frontonasal, maxillary and mandibular prominences and elongation of the optic vesciles [32], [33], phenotypes that are similar to malformations observed after Wnt/β-catenin ablation [8], [10], [15]. Furthermore, since Msx2 induces apoptosis in the developing optic cups [33], the Msx2 dose is critical for eye development and thus Msx 1/2 factors might be involved in other regulatory signalling pathways.

We have previously identified nine conserved non-coding elements (CNEs), four of which are mainly controlling *Dkk1* expression during organogenesis [34]. Among these regulatory sequences, two conserved regions, CNE114 and CNE195, function as regulatory *Dkk1* enhancers during craniofacial and optic cup development, respectively. However, the tissue specific direct regulation of *Dkk1* transcription via Tcf/Lefs and how this direct regulation is linked to additional *Dkk1* transcriptional modulators has not been addressed *in vivo*.

Here, we addressed the direct transcriptional regulation of *Dkk1* via the canonical Wnt signalling by using a combination of transgenic mouse approaches and ChIP assays. We initially identified several conserved Tcf/Lef1 binding sites within conserved *Dkk1* regulatory sequences and found that Lef1 directly binds to both, the *Dkk1* craniofacial enhancer CNE114 and optic cup enhancer CNE195 exclusively in related *Dkk1* expression domains *in vivo*. Lef1 furthermore interacts with the inactive CNE190 in the optic cups but not in craniofacial *Dkk1* expression domains. In addition, Lef1 binds to the non-conserved Tcf/Lef1 sites located within the *Dkk1* promoter. We furthermore identified conserved Msx1/2 binding sites within the conserved CNE195 optic cup enhancer and found that in addition to Lef1, Msx2 directly binds this enhancer *in vivo*. Taken together, we here unravelled a potential *Dkk1* transcriptional regulation by Lef1 and Msx1/2 transcription factors *in vivo*, using the optic cups and craniofacial *Dkk1* expression domains as important head developmental derivates.

## Materials and Methods

### Ethics statement

Animals were maintained in the approved animal facility at the university Düsseldorf and all animal work was carried out in accordance with with the relevant national guidelines for the Care and Use of Laboratory Animals. The project was approved by the ethics committee LANUV (Landesamt für Natur, Umwelt und Verbraucherschutz) located in Recklinghausen and the GMO number for the generation of transgenic mice was G207/09. Transgenic mice and nontransgenic littermates were maintained in standard laboratory conditions (12/12 hr light-dark cycle) and with full access to food and water *ad libitum*. All efforts were made to minimize suffering. For tissue collections, animals were euthanized with isoflurane followed by cervical dislocation.

### Generation of reporter constructs and site directed mutagenesis

CNE195 deletion constructs were generated by using CNE195Del1fw GCTTATTCTCTGGATTCCTA and CNE195Del1bw AAGTGATGGTCCAACACTG oligonucleotides using a full length CNE195 reporter construct as a PCR template, as described previously [31]. The resulting fragment was cloned into the existing *Dkk1* lacZ reporter construct. The same strategy was used to clone delete the larger CNE195 fragment by using CNE195Del2fw CAGTGTTGGACCATCACTT and CNE195Del2bw CTGAGCAACCAATTACTGTAC oligonucleotides. To perform site-directed mutagenesis of the CNE195 Tcf/Lef1 site, we used CNE195Mut(Tcf/Lef)fw GAATGAAACTTGAATTCGCAAGGGTCAAAAGG and CNE195Mut(Tcf/Lef)bw CCTTTTGACCCTTGCGAATTCAAGTTTCATTC oligonucleotides, spanning the Tcf/Lef binding site and performed PCR, using CNE195 in a T-vector as a template ([34]; mutagenized basepairs are underlined). The product was *DpnI* digested to reduce the amount of template background clones and the mutagenized CNE195 fragment was cloned into the *Dkk1* lacZ reporter contruct, containing a 2.7 kb *Dkk1* promotor fragment [34]. The mutagenized Tcf/Lef1 site was verified via sequencing. To perform site-directed mutagenesis of the CNE195 Msx1/2 site, we used CNE195Mut(Msx1/2)fw AAGTAATTACATTCAGATCGCAGTCCCCAAAGT and CNE195Mut(Msx1/2)bw CACTTTGGGGACTGCGATCTGAATGTAATTACTT oligonucleotides and followed the same cloning strategy as mentioned above. To perform site-directed mutagenesis of the CNE114 Tcf/Lef sites, we used CNE114Mut1(Tcf/Lef)fw GGAATTGTAGACACATGAATTCTTTGGGCATATATT and CNE114Mut1(Tcf/Lef)bw AATATATGCCCAAAGAATTCATGTGTCTACAATTCC oligonucleotides to clone this sequence into a CNE114 reporter construct as mentioned above. To generate the second mutagenesis, we used CNE114Mut2(Tcf/Lef)fw GAACTATTTGAATTCACTAGAAAAGG and CNE114Mut2(Tcf/Lef)bw CCTTTTCTAGTGAATTCAAATAGTTC oligonucleotides and performed PCR with a template containing CNE114 with the first Tcf/Lef1 site mutagenized. Otherwise, we followed the above mentioned strategy.

### Generation and genotyping of transgenic embryos

For microinjection, *lacZ* reporter constructs were isolated from the vector backbones by cutting with *SwaI/NotI*. The excised insert fragments were separated by gelelectrophoresis and purified with the QIAquick PCR Purification Kit (Qiagen). 1.5 ng/µl DNA was microinjected into male pronuclei of fertilized *B6CBAF1* mouse eggs, according to Gordon and Ruddle (1983) [35]. For the analysis of transient transgene expression, embryos were directly isolated at E12.5 and lacZ stained. DNA of embryos was isolated from Proteinase K-digested yolk sacs and tail biopsies. Genomic DNA was used as templates to identify transgenic mice with *lacZ-*specific primers GTTCCGTCATAGCGATAACGAG and CACTTACGCCAATGTCGTTATCC.

### Whole-mount-β-galactosidase staining

Embryos were fixed and stained 4–20 h as previously described [36]. LacZ stained embryos were postfixed in 0.4%PFA and paraffin embedded. 7–12 µm sections were counterstained with Eosin.

### Chromatin immunoprecipitation

Chromatin immunoprecipitation (ChIP) was performed according to the manufacturer's protocol (Sigma). For each ChIP, 10–15 E12.5 wildtype-mouse craniofacial tissues and 20–30 optic cups were dissected, pooled each and cross-linked with 1% formaldehyde at room temperature for 15 min. Whole craniofacial structures containing *Dkk1* expression domains were isolated by cutting rostral to the telencephalon and dissecting ventrally and caudal to the visible whisker buds under a stereo microscope using fine foreceps. Isolated nuclei (in 400 µl of shearing buffer) were sonicated for 12 pulses of 20 s each with a 1 min. pause in between the pulses (Duty cycle 50%, Output control 4) using a Branson Sonifier 450. The samples were sonicated on ice to shear DNA to an average size of 500 bp followed by centrifugation at 13,000rpm for 10 min. A small portion of the supernatant was now kept for the input controls. The rest was diluted 1∶2 with dilution buffer. For the immunoprecipitation, 100 µl of the sheared chromatin, mixed with 1 µg of anti-Lef1 (Millipore), 1 µg of anti-Msx1 (Sigma) and anti-Msx-2 (Santa Cruz) or control IgG antibody was used for each ChIP reaction. The immunoprecipitated DNA was analyzed by PCR. ChIP analyses was performed in at least three independent experiments. The PCR products were separated by 1.5% agarose gel electrophoresis and visualized by GelRed (Biotium). *Dkk1* CNE specific ChIP primer sequences, flanking each CNE indicated with the respective numbers and used in this study were:

ChIP25 fw: ACGGAGAGACTTCAATTAGATGCAGGC

ChIP25 bw: CCTGTAAACGGAACATGGTCAATTCCC

ChIP 114fw: GTGCACGAGAGCCAGCTGAGA

ChIP 114bw: TGGTCACTGGTGCGTCCTACG

ChIP190fw: CTGTTTAACCTTCCTGAGCC

ChIP190bw: AAATCAGCCTTCAGCTCCC

ChIP195fw: CCAGCCTCCACTAATTAAGCC

ChIP195bw: CGTCAAACAGTCCCCTACAG

ChIP Prom fw: AGTGTCAAAGTCCTCCCTGC

ChIPProm bw: ACTGCGGAACCTCAACTTCG

ChIP Exon4fw: CCGATCATCAGACTGTGCC

ChIP Exon4bw:CTTTCTGTATCCTGCAAGCC

To test for binding specificity, PCRs were performed, using primers for non-conserved 1kb flanking sequences to the analyzed CNEs.

ChIP CNE114 5′ flanking fw: TGAGGACTCATAGGTTCCAGTG

ChIP CNE114 5′ flanking bw: TTGTTCTGAGCCTCCAAGGT

ChIP CNE114 3′ flanking fw: GTGGAATCTGTGGAGACGTGA

ChIP CNE114 3′ flanking bw: ATCACAAGTTGCTGCCTCCA

ChIP CNE190 5′ flanking fw: CGGTGGACACCTAGTCACG

ChIP CNE190 5′ flanking bw: GCTTCTTGCGTTGTTCTGCTT

ChIP CNE190 3′ flanking fw: TCCCACATTTCATGTGCTGC

ChIP CNE190 3′ flanking bw: GGCTGGGACTGCTTACACAA

ChIP CNE195 5′ flanking fw: GTCTAGGGTGCAGCATCTGA

ChIP CNE195 5′ flanking bw: TGGGTTGAGGGAGCCTATTC

ChIP CNE195 3′ flanking fw: CAGCACTGTACGTGGCTAAGA

ChIP CNE195 3′ flanking bw: CCTTGGAAACTCCACCCTCC

### Electrophoretic mobility shift assay (EMSA)

EMSA assays were performed, using CNE195 oligonucleotides (Tcf/Lef)fw ATGAATGAAACTTCAAAGGGCAAGGGTCA and (Tcf/Lef)bw TGACCCTTGCCCTTTGAAGTTTCATTCAT nucleotides. For EMSA assays with the mutated Lef1 site, we used CNE195Mut(TCF/Lef)fw ATGAATGAAACTTGAATTCGCAAGGGTCA and CNE195Mut(TCF/Lef)bw TGACCCTTGCGAATTCAAGTTTCATTCAT, as described above for the generation of Tcf/Lef1 mutagenized transgenic mice. 1 nm single stranded oligonucleotides were denaturated for 5 min at 95°C and annealed by slow reduction to room temperature. The resulting double stranded DNA was labelled with radioactive γ^32^P (Amersham) by T4-polynucleotid kinase (NEB) for 30 min at 37°C. After determination using centilation counter, the radioactive labelled nucleotides were isolated, using a Nucleotide removal kit (Qiagen). Lef1 protein was produced, using Lef1 with the amino acids 1-397 in a pGEX4T-1 vector was a gift from Thomas Theil. Recombinant Lef1/GST fusion proteins were pepared as described [37]. The Lef1 protein was isolated from the GST ancor by thrombin and 100 ng were incubated 30 min. on ice in 20 mM Hepes, pH7.9; 60 mM KCl; 1 mM EDTA, pH8.0; 1 mM DTT; 5 mM MgCl_2_; 10% glycerol and 1 ug poly(dI-dC) in the presence of 10.000 cpm of end-labelled oligonucleotides or unlabelled-oligos as a binding reaction. Electrophoresis was performed through 5% native polyacrylamide gels in 0.5xTBE at 4°C. Radioactive signals were detected by phosphoimager plates and analysed by the use of a phosphoimager.

### In situ hybridisation

After dissection in ice cold PBS, embryos were fixed over night in 4%PFA. *In situ* hybridization of whole mount embryos, using digoxigenin-labelled riboprobes was performed as previously described [34]. For in situ hybridizations on sections, embryos were dehydrated with 70, 80, 90 and 100%ethanol, respectively for 2 hours. After an overnight incubation in 1-butanol, embryos were transferred into paraffin for embedding. *In situ* hybridizations on paraffin sections were performed on 7 µm frontal sections as previously described [34]. Antisense RNA riboprobes were prepared by reverse transcription from linearized plasmids containing complete or partial sequences of *Dkk1, Lef1*, *Msx1* and *Msx2* genes.

### Identification of conserved transcription factor binding sites

Conserved transcription factor binding sites were identified using several data bases (http://ecrbrowser.dcode.org; http://ensembl.org; http://genome.lbl.gov/vista/index.shtml).

### Real time PCR

Varying LiCl concentrations were added to MEFs that were analyzed between passage 7 and 10. For preparation of RNA, MEFs were harvested and RNA isolation was performed directly. After RNA extraction, including DNAseI digestion from the homogenized tissue (Qiagen), RNA was reverse transcribed, according the manufacture's protocol (Qiagen). TaqMan Gene Expression Assays (Applied Biosystems) were performed with Real Time Ready master mix (Applied Biosystems) in a StepOne (Applied Biosystems) thermal cycler, according to the manufacture's protocol. Relative fold expression changes of *Dkk1* of untreated and LiCl treated MEFs were calculated using the comparative -ΔCt method. Normalization was performed using the housekeeping gene *GAPDH*. Standard errors were computed from at least three genotypes.

### PCR band intensity quantifications and statistical analysis

After PCR amplification, band intensities were quantified using the imageJ software (http://imagej.nih.gov/ij/). At least three experiments were performed and tested for significance, using a students T-test.

## Results

### The *Dkk1* craniofacial and optic cup enhancers contain conserved Tcf/Lef1 sites

**Figure 1:**
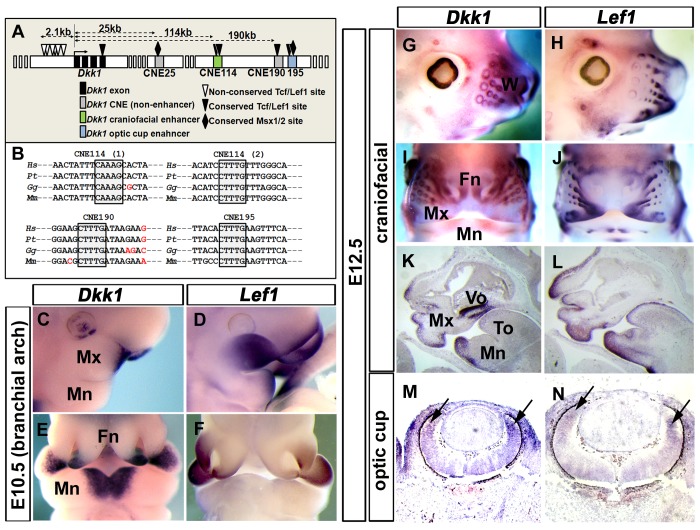
Sequence conservation of Tcf/Lef1 binding sites at the *Dkk1* locus and coexpression of *Lef1* and *Dkk1*. (A) Schematic representation of the mouse *Dkk1* locus, indicating Tcf/Lef1(triangles) and Msx1/2 (diamonds) binding sites addressed in this study. The *Dkk1* transcriptional start (arrow) and *Dkk1* conserved non-coding elements (CNEs; filled boxes) and their distances to the *Dkk1* transcriptional start localization are indicated. (B) Sequence alignment of human (*Hs*), chimpanzee (*Pt*), chicken (*Gg*) and mouse (*Mm*) CNE114, CNE190 and CNE195 conserved sequences revealing a 100% sequence identity in the aligned vertebrate Tcf/Lef1 core binding sites (highlighted in grey). (C-N) Comparative expression analysis of *Dkk1* (C, E, G, I, K, M) and *Lef1* (D, F, H, J, L, N) in E10.5 branchial arch (C-F) and E12.5 craniofacial expression domains (G-L) using whole mount (C-J) and saggital sectioned (K+L) embryos. Maxillary (Mx), mandibular (Mn) and frontonasal (Fn), vomeronasal (Vo) and tongue (To) domains are indicated. *Dkk1* expression overlaps with *Lef1* expression at E10.5 in the frontonasal mass but not in other 1^st^ branchial arch derivates (C-F). Expressions broadly overlap at E12.5 craniofacial expression domains (G-L). *Dkk1* expression is very similar to *Lef1* expression in the anterior retina of E12.5 optic cups (as indicated by arrows in M+N).

Initially, we addressed the ability of the canonical Wnt signalling to activate *Dkk1* expression using mouse embryonic fibroblasts (MEFs). We found that Wnt/β catenin signalling stimulation by a LiCl treatment caused a 4 fold induction of the *Dkk1* expression level (S1A Fig.). These data suggested that the canonical Wnt signalling activity is sufficient to promote *Dkk1* expression *in vitro*. Therefore, we hypothesized that the *Dkk1* transcription is directly regulated by the canonical Wnt signalling. To address this, we searched for conserved Tcf/Lef1 binding sites within the previously identified conserved non-coding elements (CNEs) at the *Dkk1* locus [34]. We found four conserved Tcf/Lef1 binding sites, residing within three of the *Dkk1* CNEs (Fig. 1A, black triangles). A detailed sequence analysis of several vertebrate genomes revealed that these Tcf/Lef1 binding sites share a 100% sequence conservation within their core binding sequences (Fig. 1B). However, we could not identify homologous binding sites in lower vertebrate taxa such as fish and amphibians, suggesting that these sequences are not deeply conserved. Most importantly, two of the conserved sites are localized within the *Dkk1* craniofacial regulatory enhancer CNE114 and one in the optic cup enhancer CNE195 [34]. In addition to the conserved Tcf/Lef1 binding sites, several Tcf/Lef1 sites reside within in the mouse *Dkk1* promoter, in homology to the binding sequences previously identified in human (Fig. 1A, white triangles; [28]–[30]). To dissect out, whether Lef1 could potentially regulate *Dkk1*, we next characterized the dynamics of *Lef1* and *Dkk1* expressions within the branchial arches and their craniofacial derivates, according to [9], [12], [13], [14], [38], [39]. We found that *Dkk1* expression is overlapping with the anterior *Lef1* expression domain in the maxillary region of the 1^st^ brachial arch (Fig. 1C–F) and the frontonasal mass at E10.5 (Fig. 1E+F), however, the overall *Lef1* expression was much broader in the maxillary domain. In addition, *Lef1* expression was very weak in the mandibular mesenchyme (Mn), where *Dkk1* was characteristically expressed (Fig. 1E+F). Interestingly, when the 1^st^ brachial arch progenitor tissues differentiated into defined craniofacial derivates at E12.5, *Lef1* was coexpressed with *Dkk1* in a broad fashion including various craniofacial domains, such as the whisker hair bud mesenchyme (W), the frontonasal mass (Fn) and mandibular domains (Fig. 1G–J). As revealed by craniofacial sagittal sections, *Lef1* was expressed very similar to *Dkk1* in mesenchymal regions, such as the frontonasal mass, mandibular and maxillar domains (Fig. 1K+L). However, *Lef1* expression is absent in the mesenchyme below the vomeronasal organ, in which *Dkk1* is characteristically expressed. Together, these data suggest that *Dkk1* and *Lef1* expressions got synchronized after 1^st^ branchial arch progenitor differentiation into their craniofacial derivates. We next characterized the expression patterns of *Dkk1* and *Lef1* in the optic cups, in which CNE195 acts as a *Dkk1* regulatory enhancer [34]. In the developing eyes, *Dkk1* is expressed in the dorsal and ventral optic vesicles at earlier stages and the anterior retina and the retinal pigment epithelium at E12.5 (arrows in Fig. 1M; [9], [10], [40]), very similar to *Lef1* (arrows in Fig. 1N; [41]). However, *Lef1* expression itself is positively regulated by the Wnt signalling and thus might explain the coexpression in the above mentioned areas. We suggest that Lef1 mediated Wnt signalling might regulate *Dkk1* expression by direct interaction with its conserved binding sites, located within the *Dkk1* craniofacial enhancer (CNE114) and the optic cup enhancer (CNE195).

### Lef1 interacts with its non conserved Tcf/Lef1 promotor binding sites

In addition to the conserved Tcf/Lef1 binding sites residing within conserved regions, we found that several Tcf/Lef1 sites are localized within the mouse *Dkk1* promoter, in homology to the target sequences previously identified in human (Fig. 1A, white triangles; [28]–[30]). To test the regulatory relevance of the *Dkk1* promoter itself, we performed ChIP assays, using a Lef1 specific antibody on crosslinked E12.5 craniofacial and optic cup DNA. We found that Lef1 interacts with its *Dkk1* promoter binding sites (S1B Fig.) in contrast to a conserved *Dkk1* exonic Tcf/Lef1 binding site (S1C Fig.), suggesting that the interaction between the *Dkk1* promoter and Lef1 is specific. In addition, we found that Lef1 interferes with its Tcf/Lef1 *Dkk1* promoter binding sites in the optic cups (S1D+E Fig.). Together, these data suggest that Lef1 commonly interacts with the *Dkk1* promoter in the optic cups and craniofacial domains, although this fragment alone is not sufficient to promote tissue specific *Dkk1* expression during embryonic development [34].

### The CNE195 Tcf/Lef1 binding site is required for *Dkk1* reporter activation in the optic cups

**Figure 2:**
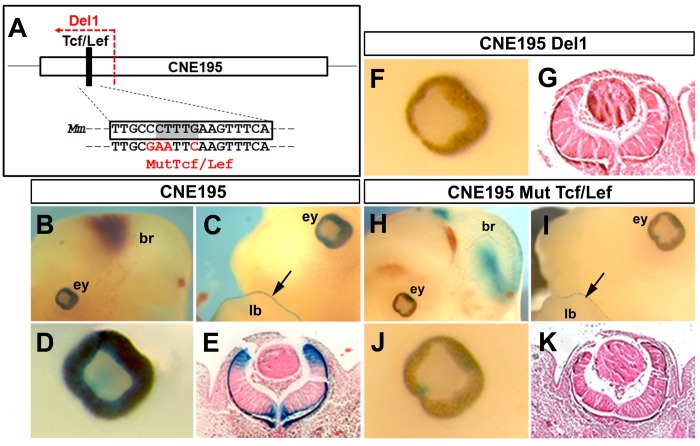
The CNE195 Tcf/Lef1 binding site is required for *Dkk1* retinal activation *in vivo*. (A) Schematic representation of the mouse CNE195 locus, indicating the sites of the CNE195 5′ depletion (red arrow), and point mutagenesis of the mouse Tcf/Lef1 binding site performed in Fig. 2 experiments. The Tcf/Lef1 core binding site is highlighted in grey; exchanged bases for the generation of mutagenized transgenic embryos are marked in red. (B-K) X-gal stained whole mount (B, C, D, F, H, I, J) and transverse sectioned (E, G, K) transient E12.5 transgenic optic cups after pronuclear injection of the endogenous CNE195 (B-E), the 5′deleted CNE195 (F+G) and the mutagenized Tcf/Lef1 CNE195 construct (H-K). Transgene expression under control of the CNE195 reporter construct promotes reporter activity in the optic cups (ey), limb buds (lb) and weakly in the brain (br) (B+C). Reporter activity is evident in the anterior retina and the basal region where the retina enters the optic stalk and the retinal pigment epithelium (D+E). Deletion of the 5′ portion of CNE195 results in a loss of reporter gene activity throughout the optic cups (F+G). Point mutagenesis of the Tcf/Lef1 site results in a maintenance of reporter expression in the brain and the limb buds (H+I) and a drastic reduction of optic cup reporter gene expression in transgenic embryos at E12.5 (J). Reporter gene activity was not detectable in the retina (K). Together, eight transgenic embryos were obtained for the CNE195 construct, of which four embryos exhibited identical staining in the optic cups, the brain and metanephros at E12.5 [34]. In total six embryos were obtained after pronuclear injection of the 5′deleted CNE195 construct, of which three expressed the transgene in brain and metanephros but not the optic cups. In total, seven embryos were obtained after injection of point mutagenized CNE195, of which four exhibited a loss of reporter expression exclusively in the optic cups. The remaining transgenic embryos presented showed no expression.

We next focused on the transcriptional regulation of *Dkk1* via the conserved CNE195 Tcf/Lef1 binding site. We used a CNE195 *Dkk1* reporter construct, containing of a 2.1kb *Dkk1* promoter fragment, linked to a lacZ reporter sequence and the full CNE195 sequence that completely mirrored *Dkk1* expression in the developing optic cups and limb buds in all four transient transgenic embryos at E12.5 [Fig. 2B–E; n = 4/8; table 1]. We modified the full length CNE195 sequence by the deletion of 96 bp of the 5′ region, containing the conserved Tcf/Lef1 site (Fig. 2A). We used this modified CNE195 reporter construct (CNE195Del1) to address the regulatory ability by analyzing transient reporter expression in transgenic embryos at E12.5. Transient reporter activity was present in *Dkk1* specific expression domains such as the brain and metanephros but was lost within the apical ectodermal ridge (AER) (n = 3/6; table 1; S2A, E and I Fig.). Most importantly, in contrast to the CNE195 reporter activity in the anterior retina and the retinal pigment epithelium (Fig. 2B–E; [34]), none of the embryos carrying the CNE195Del1 transgene exhibited a reporter gene activity in the optic cups (Fig. 2F+G). In order to test, whether the Tcf/Lef1 binding site localized within the deleted fragment is required for reporter gene expression in the optic cups, we generated C/G, C/A, T/A, G/C exchanges in the CNE195 Tcf/Lef1 core binding site to diminish potential Tcf/Lefs binding, as previously shown (Fig. 2A; [42]). Transient transgenic embryos carrying the MutTcf/Lef1 construct showed reporter activity in the brain, limbs and the metanephros (n = 4/7; Fig. 2H+I; Fig. S2B, F and J). Strikingly, reporter activity in the retina was completely absent in these transgenic embryos, except a weak residual activity in the pigment epithelium at E12.5 (Fig. 2J+K). Together, these data demonstrate that the conserved Tcf/Lef1 binding site within CNE195 is required for retinal *Dkk1* reporter expression.

**Table 1:** *Dkk1* reporter constructs and transient transgenic embryos analyzed throughout this study.

	Transgenic embryos	lacZ expression	Optic cups	Craniofacial (wh, fm, mx, mn)	brain	limbs	metanephros
CNE195 wt	8	4	4	-	4	4	4
CNE195 Del1	6	3	-	-	3	-	3
CNE195 Mut Tcf/Lef1	7	4	-	-	4	4	4
CNE195 Del2	5	2	-	-	2	-	-
CNE195 mut Msx1/2	4	2	-	-	2	2	2
CNE114 wt	4	3	-	3	3	-	3
CNE114 Del	6	5	-	5 (fm, mx, mn)	5	-	5
CNE114 Tcf/Lef1 Mut2	4	3	-	3 (wh, fm+++, mx+++, mn)	3	-	3
CNE114 Tcf/Lef1 Mut1,2	3	2	-	-	2	-	2

For each analyzed *Dkk1* reporter construct, the total number of achieved transgenic E12.5 embryos and the respective amount of lacZ expressing embryos is given. Of those, the number of identical tissue specific expressions in optic cups, craniofacial domains, the brain, limbs and metanephros is given. Wh =  wisker hairbuds; fm =  frontonasal mass; mx =  maxillar and mn =  mandibular domains. Ectopic expression within a given domain is indicated with “+++”.

### Lef1 interferes with the CNE195 eye enhancer

**Figure 3:**
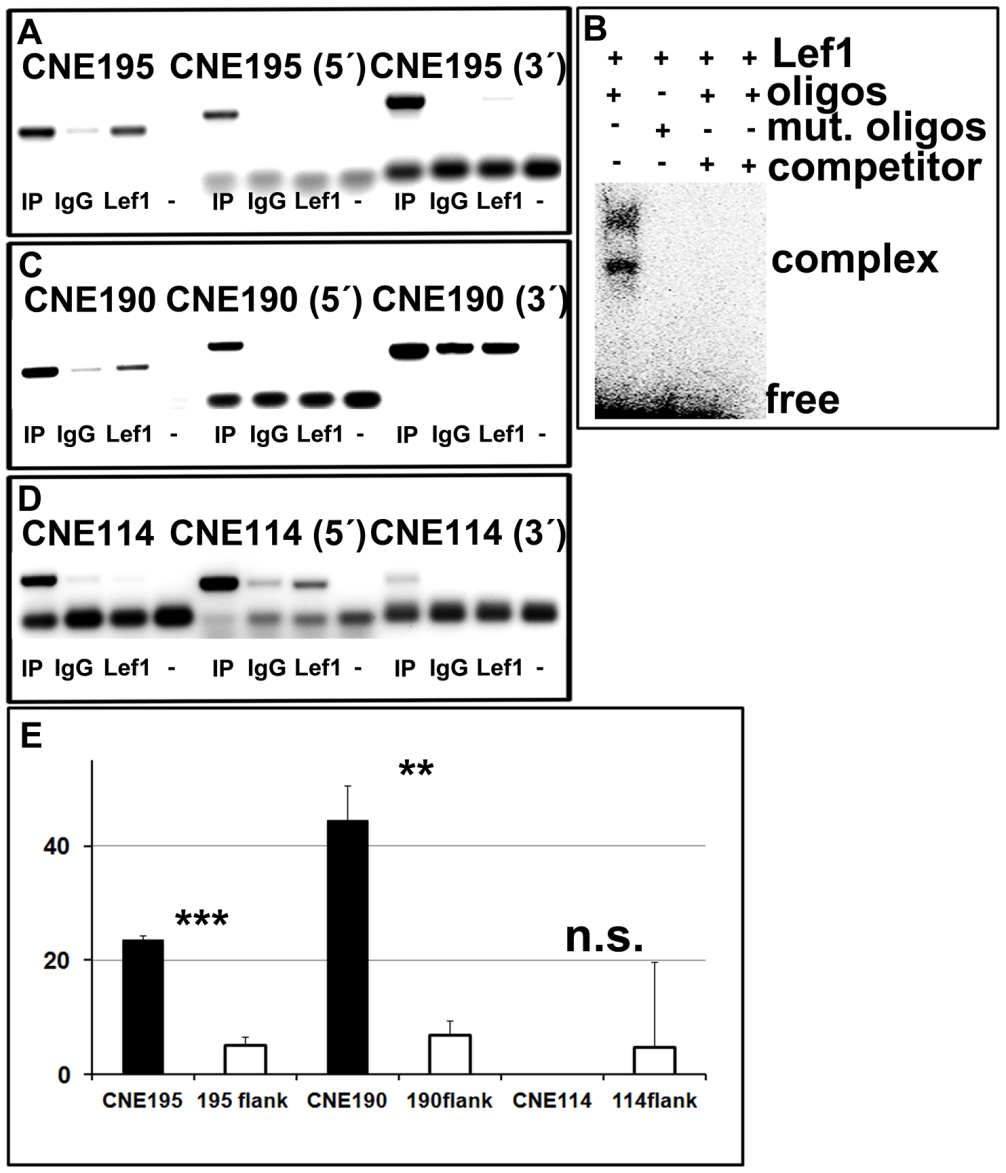
Lef1 interacts with CNE195 via its conserved binding site. (A, C, D) ChIP assays on crosslinked E12.5 optic cup DNA, using a Lef1 specific antibody. An IgG antibody was used as a control. Input DNA (IP) and immunoprecipitates were analyzed by PCR using CNE195, 190 and 114 specific primers, as indicated. For each ChIP analysis, 1 kb flanking primers were used for amplification to test for binding specificity of the Lef1 binding to the analyzed CNE. (A+C) Lef1 interacts with both, CNE195 and CNE190 in the optic cups in contrast to the flanking control ChIP PCRs. (B) Electrophoretic mobility shift assays, using isolated Lef1 protein and radioactively labelled CNE195 Tcf/Lef1 oligonucleotides. Lef1 directly binds to the CNE195 binding site. (D) Lef1 does not bind the craniofacial enhancer CNE114 in the optic cups. (E) Quantification of the ChIP results presented in (A, C, E). Bars represent the average band intensity related to the input signal intensity in percent. Lef1 binding to CNE195 (p-value  = 0,00064) and CNE190 (p-value  = 0,0066) is highly significant in the optic cups but CNE114 is not (p-value  = 0,387). Representative results of at least three independently performed ChIP assays or EMSAs are presented in this figure.

To unravel, whether the loss of retinal reporter expression after mutagenesis of the Tcf/Lef1 site correlates with Lef1 binding to the CNE195 eye enhancer *in vivo*, we performed chromatin immunoprecipitation (ChIP). We used a Lef1 specific antibody on crosslinked DNA-protein complexes from E12.5 isolated optic cup tissue. ChIP analysis of this tissue using CNE195 specific oligonucelotides revealed that Lef1 indeed interacts specifically with CNE195 in contrast to the IgG control and CNE195 1 kb upstream or downstream sequences (Fig. 3A). To verify this result, we performed electrophoretic mobility shift assays (EMSA). The Lef1 protein caused a reduced mobility of a labelled 32 bp oligo of CNE195, containing the Tcf/Lef1 site at its centre in contrast to the oligonucleotides containing the mutated Lef1 binding site (Fig. 3B).

We next tested, whether CNE190, which also contains a highly conserved Tcf/Lef1 binding site (Fig. 1), interacts with Lef1 in the optic cups, using again the ChIP assay approach. Interestingly, we observed that in addition to CNE195, Lef1 binds to CNE190 in the optic cups, as well (Fig. 3C). Since CNE190 alone does not function as a regulatory enhancer, we hypothesized, that Lef1 might generally interact with its conserved binding sites present in other *Dkk1* CNEs. Therefore, we next tested, whether Lef1 interacts with the craniofacial enhancer CNE114 in the optic cups, since this enhancer also contains two conserved Tcf/Lef1 sites (see above). However, the Lef1 antibody did not precipitate isolated E12.5 crosslinked optic cup DNA (Fig. 3D). Quantifications of Lef1 binding to the conserved binding sites revealed that Lef1 binding to CNE195 and CNE190, but not CNE114 was highly significant and specific to the analyzed CNE regions (Fig. 3E). Together, these data reveal that the CNE195 reporter expression correlates with potential Lef1 binding to CNE195 in the optic cups. In addition, Lef1 potentially interacts with the inactive CNE190 but not the craniofacial enhancer CNE114 in this tissue.

### *Dkk1* CNE195 reporter expression correlates with binding of Msx2 in the optic cups

**Figure 4:**
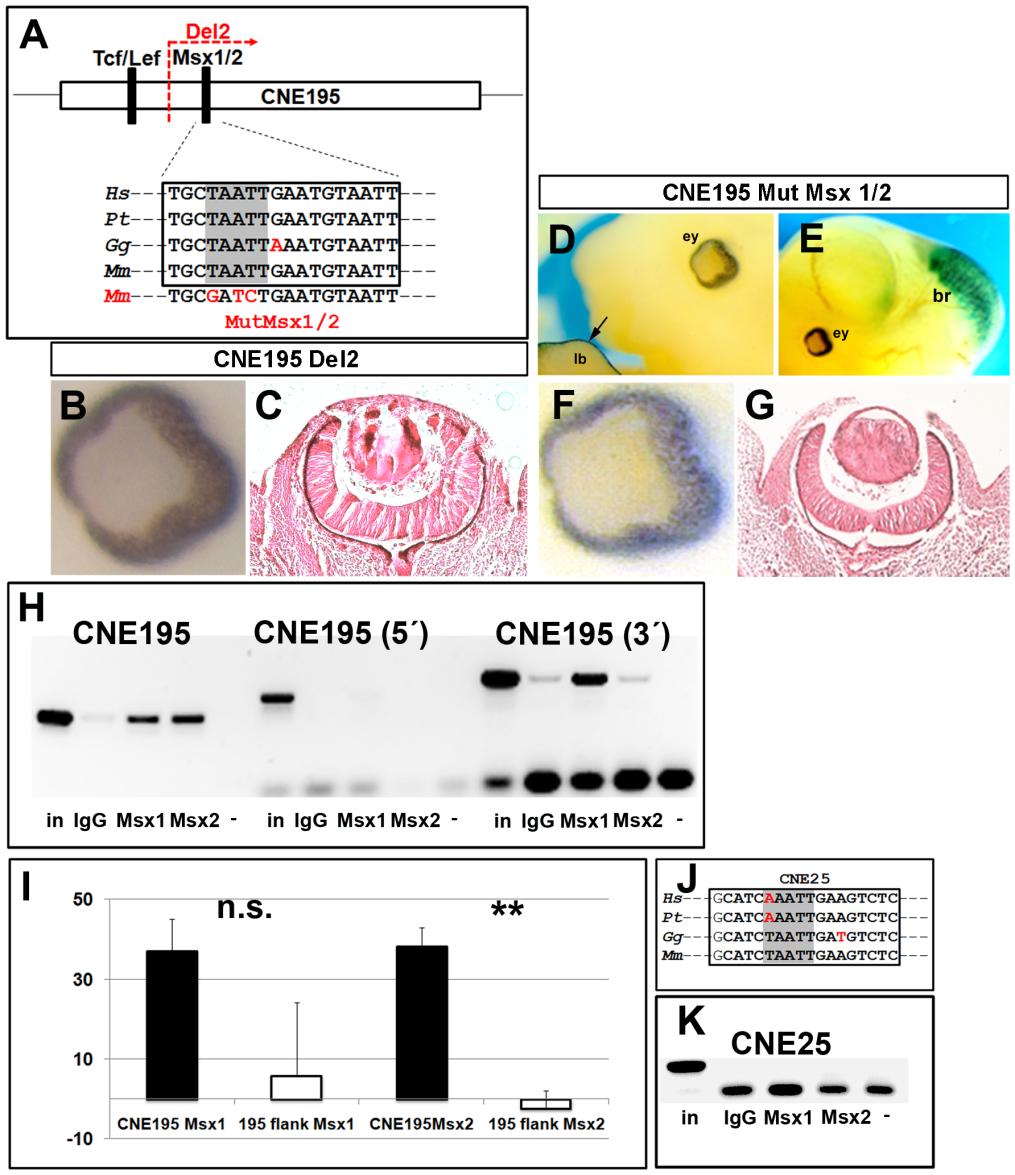
CNE195 reporter activity correlates with binding of Msx2 to its conserved binding site. (A) Schematic representation of CNE195, containing the conserved Tcf/Lef1 binding site, the Msx1/2 binding site and the site used for deletion of the 3′ CNE195 fragment. The core Msx1/2 binding site is highlighted in grey and the related exchanged bases for mutagenesis performed in this figure are highlighted in red. Sequence alignment of human (*Hs*), chimpanzee (*Pt*), chicken (*Gg*) and mouse (*Mm*) CNE195 conserved sequences revealing 100% sequence identity in the aligned vertebrate Msx1/2 core binding site loci (highlighted in grey). (B, D, E, F) whole mount and (C+G) sectioned optic cups of E12.5 X-gal stained transient transgenic embryos after pronuclear injection of the 3′ deleted CNE195 (B+C) or the Msx1/2 mutagenized reporter construct (D-G). Transient reporter expression of transgenic embryos using eather constructs was present in the brain and limb buds (D+E) but lost in the optic cups (C+G). (H) ChIP assays on crosslinked E12.5 optic cup DNA using antibodies against Msx1 and Msx2. Input DNA (IP) and immunoprecipitates were analyzed by PCRs with CNE195 specific and 1 kb 5′ or 1kb 3′ flanking primers as indicated. (I) Quantification of the band intensity related to the input signal intensity in percent revealed a highly significant binding of Msx2 but not Msx1 to CNE195 (p values  = 0,122 (Msx1); 0,00089 (Msx2)). (J) Sequence alignment of human (*Hs*), chimpanzee (*Pt*), chicken (*Gg*) and mouse (*Mm*) CNE25 Msx1/2 conserved binding site revealing high sequence conservation of the core Msx1/2 binding site (highlighted in grey). (K) ChIP assays using CNE25 specific primers. Neither Msx1, nor Msx2 interferes with CNE25 in the optic cups. In total, five transgenic embryos were obtained after injection of the depleted 3′ CNE195 reporter construct. Two of these exhibited a loss of reporter expression. Four transgenic embryos were obtained after injection of the CNE195 Msx1/2 mutagenized construct, of which two exhibited a drastic loss of retinal reporter expression exclusively within the optic cups. The remaining transgenic embryos presented showed no expression. Representative results of at least three independently performed ChIP assays are presented in this figure.

Since Lef1 binding to its target sequences is associated with the simultaneous binding of additional factors to adjacent sites [25], we hypothesized that other factors might regulate *Dkk1* transcription in the optic cups via CNE195. To address this, we deleted the complementary larger 3′ portion of CNE195 tested above and used this construct for the generation of transient transgenic embryos (Fig. 4A). Reporter activity was evident in the brain but lost in most of the other CNE195 activity domains, including the limbs and the metanephros at E12.5 (n = 2/5; S2C, G and K Fig.). Most importantly, we found that deletion of this sequence caused a complete loss of reporter gene expression in the optic cups in all transgenic embryos (Fig. 4B+C). These data suggest that this portion of CNE195 must contain other transcription factor binding sites, controlling *Dkk1* expression in the optic cups. Therefore, we screened this CNE195 portion to identify additional conserved binding sites and found that the Msx1/2 core binding site is highly conserved in vertebrates with a 100% sequence conservation within CNE195 (Fig. 4A). *Msx1* and *Msx2* gene ablations result in strikingly similar optic cup phenotypes compared to a reduction of the Dkk1 level [43], [19] and *Msx1/2* genes are coexpressed with *Dkk1* in the optic cups [4], [10]. We therefore directly tested, whether the Msx1/2 binding site is required for CNE195 reporter activity *in vivo* and generated T/G, A/T, T/C base pair exchanges in the Msx1/2 core binding site (Fig. 4A; red characters). We used the according Msx1/2 mutated CNE195 reporter construct for pronuclear injections. Transient reporter gene expression was evident in CNE195 activity domains such as limbs, brain and metanephros (n = 2/4; Fig. 4D+E; S2D, H and L Fig.), however, reporter gene activity was undetectable in the retina in all transgenic embryos at E12.5 (Fig. 4F+G). Although we cannot completely rule out a loss of reporter expression in the retinal pigment epithelium, these data show that the conserved Msx1/2 binding site is essential for *Dkk1* reporter activity in the developing retina. We next addressed, whether the loss of reporter gene expression correlates with Msx1 and/or Msx2 binding to this enhancer. Because Msx1 and Msx2 transcription factors share common core binding nucleotides [44], we performed ChIP assays using either Msx1 or Msx2 specific antibodies on crosslinked DNA from E12.5 isolated optic cups. Our ChIP assays revealed that predominantly Msx2 binds CNE195 (Fig. 4H+I). However, Msx1 resulted in a significant signal in the 3′ flanking amplification, possibly due to a non-conserved Msx1/2 binding site we identified within this sequence (data not shown). Because we identified a conserved Msx1/2 binding site within CNE25 with 100% sequence identity in mouse and chicken but not *Pan troglodytes* or *Homo sapiens* (Fig. 4J), we tested, whether the related mouse site is bound by Msx1/2 transcription factors and performed ChIP assays on crosslinked E12.5 optic cup DNA, using CNE25 specific oligonucleotides. However, neither Msx1 nor Msx2 bound the CNE25 in the optic cups at this developmental stage (Fig. 4K). These data show that in the optic cups, CNE195 *Dkk1* reporter gene expression correlates with Msx2 (but not Msx1) interaction with its conserved CNE195 binding site.

### Two CNE114 Tcf/Lef1 binding sites are critical for *Dkk1* craniofacial reporter activity

**Figure 5:**
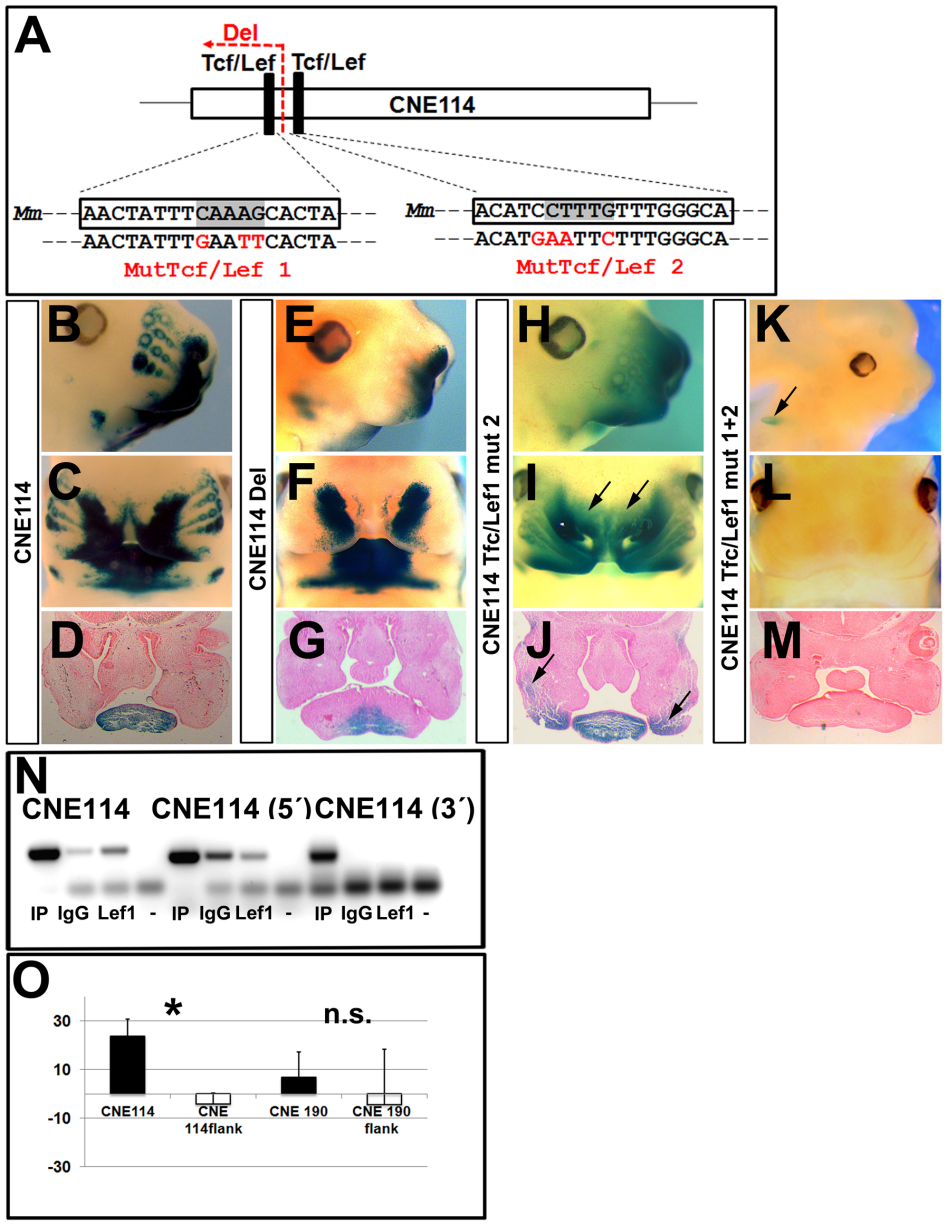
Craniofacial *Dkk1* reporter activation correlates with Lef1 binding to its conserved CNE114 binding sites. (A) Schematic representation of the CNE114 conserved Tcf/Lef1 binding sites. The Tcf/Lef1 core binding sequences are highlighted in grey and Tcf/Lef1 exchanged bases are indicated in red. (B-M) Transient transgene expression in E12.5 transgenic embryos after pronuclear injection of the CNE114 (B-D), the 5′ deleted CNE114 (E-G), the Tcf/Lef1 site2 mutagenized CNE114 (H-J), or the Tcf/Lef1 site1 and Tcf/Lef1 site2 mutagenized reporter constructs (K-M). (B, E, H, K) side views, (C, F, I, L) frontal views and transverse sectioned lacZ stained embryos (D, G, J, M). In comparison to the unmodified CNE114 (B+C), reporter expression is lost in the whisker hair bud mesenchyme but maintained in nasal domains and mandibular domains after deletion of the 5′CNE114 region (E+F). The mutagenized Tcf/Lef1 site2 leads to an overall reduction in reporter expression in the whisker hair bud mesenchyme and the mandibular expression domains (H+I) and expanded transgene expression into maxillar mesenchymal domains (arrows in J). Simultaneous mutagenesis of both Tcf/Lef1 sites results to a complete loss of reporter expression in craniofacial domains (K-M; arrows in “K” point to the reporter expression in the otic vesicles). In total four transgenic embryos were obtained with the CNE114 construct, of which three exhibited identical staining. Together, six transgenic embryos were obtained after injection of the 5′ deleted CNE114. Five of these exhibited identical expression as presented in this figure. Together, four transgenic embryos were obtained after mutagenesis of CNE114 Tcf/Lef1 binding site 2, of which three exhibited identical staining indicated in (H-J). In total, three transgenic embryos were obtained after point mutagenesis in both Tcf/Lef1 binding sites, of which two exhibited a loss of reporter expression in craniofacial expression domains. The remaining transgenic embryos presented showed no expression. (N) ChIP assays with E12.5 crosslinked craniofacial DNA, using a Lef1 antibody, as indicated. Input DNA (IP) and IgG controls and oligonucleotides flanking the CNE114 that were used for amplification are indicated. Lef1 directly binds the conserved CNE114 Tcf/Lef1 binding sites. Lef1 did not result in a positive signal, when PCRs were performed, using primers specific to 1kb CNE114 5′ and 3′ flanking sequences. (O) Quantification of the ChIP results represented by the average band intensity related to the input signal in percent reveals a significant binding of Lef1 to its CNE114 binding sites (p value  = 0,013). Representative results of at least three independently performed ChIP assays are presented in this figure.

In addition to the conserved Tcf/Lef1 sites in the optic cup enhancer CNE195, we identified two conserved vertebrate Tcf/Lef1 binding sites within the craniofacial *Dkk1* enhancer CNE114 (Fig. 1). Therefore, we used this tissue to dissect out the *Dkk1* transcriptional regulation via Tcf/Lef1 in craniofacial domains. We initially addressed, whether the 5′ portion of CNE114 containing one of the two conserved Tcf/Lef1 binding sites (Tcf/Lef no. 1) is required for *Dkk1* reporter activity in craniofacial tissues and deleted a 166 bp fragment of CNE114. Using this construct, reporter activity was evident in *Dkk1* expressing craniofacial expression domains such as the frontonasal mass and the mandibular and maxillar domains, identical to the unmodified CNE114 reporter expression, however, reporter activity within the whisker hair bud mesenchyme was absent (Fig. 5E–G; n = 3/4 and B–D; n = 5/6). Furthermore, transient reporter activity was maintained in the brain (S3A+B Fig.). Next, we analyzed whether the adjacent Tcf/Lef1 binding site is required for craniofacial reporter activity. Transient transgenic embryos carrying a point mutation in the Tcf/Lef1 (no. 2) binding site generally exhibited reporter expression in craniofacial domains at E12.5 (Fig. 5H+I; n = 3/4). However, as revealed by transverse sections, transgene expression appeared diffuse and expanded within the maxillar mesenchymal expression domains and the frontonasal mass in these embryos (arrows in Fig. 5I+J). Furthermore, the overall expression in the brain was reduced but generally maintained (S3C+D Fig.). Together, these data show that the Tcf/Lef1 site no. 1 is required for *Dkk1* reporter expression in the whisker hair bud mesenchyme and the Tcf/Lef1 site (no2.) is involved in repressing the expression within maxillar mesenchymal and frontonasal domains. We therefore generated point mutations in both Tcf/Lef1 sites simultaneously and tested the impact on craniofacial *Dkk1* reporter expression in combination. By using this construct, transient reporter expression was completely absent in all craniofacial domains at E12.5 (n = 2/3; Fig. 5K–M). However, reporter expression in other head derivates was reduced, we found weak expression in the otic vesicles and the midbrain (arrow in Figs. 5K; S3E–I). Together, these data suggest that the CNE114 Tcf/Lef1 sites are together required for the *Dkk1* transcriptional activation in craniofacial expression domains. We next analyzed, whether Lef1 interacts with its conserved binding sites present in CNE114. To do so, we dissected E12.5 craniofacial tissues, containing the *Dkk1* specific expression areas and performed ChIP analysis, using a Lef1 specific antibody on crosslinked DNA. Indeed, we found that Lef1 specifically interacts with CNE114 (Fig. 5N+O). However, unlike in the optic cups, we did not observe that Lef1 binds CNE190 in craniofacial domains (Fig. 5O). Together these data show that in craniofacial domains, the CNE114 *Dkk1* reporter activity correlates with a Lef1 interaction with its conserved CNE114 binding sites.

## Discussion

**Figure 6:**
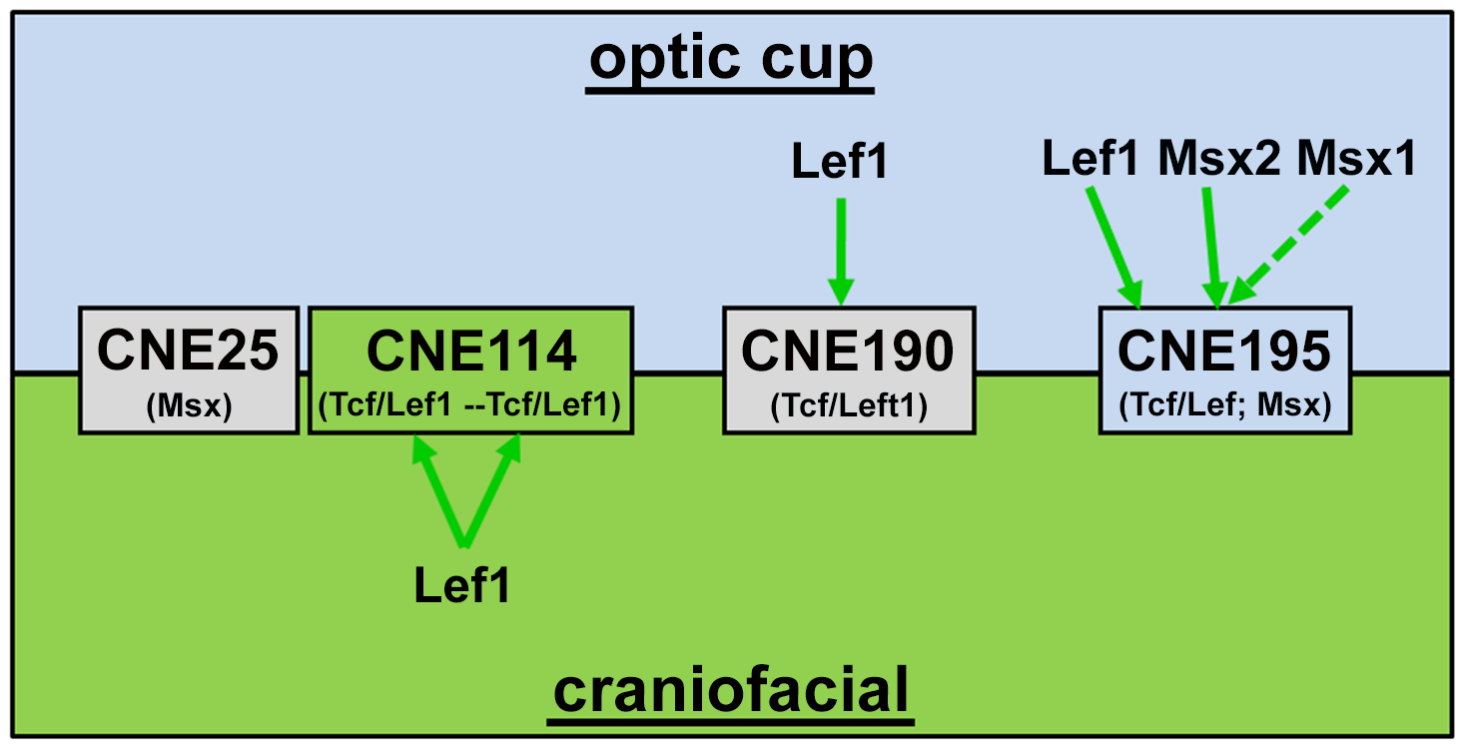
Model: Differential binding of *Dkk1* by Lef1 and Msx1/2 in the optic cups and craniofacial domains via CNEs. Four conserved *Dkk1* regulatory elements (CNE25, CNE114, CNE190 and CNE195) are characteristically recruited by Lef1, Msx1 and Msx2 transcription factors within the optic cups and craniofacial domains: Lef1 binds both, the CNE195 optic cup enhancer and the CNE114 craniofacial enhancer. Lef1 furthermore interacts with the conserved CNE190 locus in the optic cups. In addition, Msx2 but not Msx1 binds CNE195 within the optic cups. Blue, green and grey boxes indicate the conserved non-coding elements, representing the optic cup and craniofacial enhancers and non- enhancer conserved regions, respectively.

In this study, we addressed the differential regulation of *Dkk1* expression by Lef1 and Msx1/2 transcription factors using the optic cups and craniofacial tissues (summarized in Fig. 6). We found that the *Dkk1* reporter activity correlates with the differential binding of these factors *in vivo* but the binding capacities do not follow common characteristics: Lef1 might regulate *Dkk1* by interaction with its conserved binding sites, localized within both, the craniofacial (CNE114) and optic cup (CNE195) enhancers, respectively. In craniofacial expression domains, the simultaneous interaction with both sites is mandatory for the general craniofacial *Dkk1* transcriptional activation. Interestingly, the two craniofacial Tcf/Lef1 sites are commonly bound in the whisker hair bud mesenchyme, but, however, the Tcf/Lef1 site no. 2 is specifically required for the restriction of maxillar mesenchymal expression domains.

In addition to Lef1, Msx2 but not Msx1 binds CNE195 in the optic cups. Together, our summary suggests a direct negative Wnt-Dkk1 feedback loop, in which the active Wnt signalling limits its own activity by promoting *Dkk1* expression via tissue specific *Dkk1* enhancers. In the optic cups, Msx2 in addition to Lef1 binds the *Dkk1* enhancer most likely to favour *Dkk1* expression in the expense of Wnt signalling.

The described data demonstrate that Lef1 characteristically binds its *Dkk1* enhancer CNE transcription factor binding sites in tissues, in which the respective enhancer is active, since Lef1 does not target CNE114 in the optic cups or CNE195 in craniofacial tissues even though Lef1 is co-expressed with *Dkk1* in these domains (Fig. 1). These results support the idea that Lef1 binding is strongly dependent on additional tissue specific transcription factors that bind to adjacent sites and thereby enabling Lef1 recruitment. This might be facilitated by either a direct interaction of other factors and/or by the induction of conformational chromatin changes. We indeed could identify several conserved transcription factor binding sites for key eye regulator proteins within CNE195, such as Pax6 and Pax2/5/8 (own observation). The presence of these proteins in the optic cups but not craniofacial domains would therefore specify Lef1 binding to CNE195 in the optic cups. In addition, the binding of a conserved *Dkk1* regulatory region by Lef1 does is not *per se* result in a transcriptional activation of *Dkk1*, since e.g. CNE190 is not of relevance for *Dkk1* expression in the optic cups.

### Regulation of *Dkk1* and implications for optic cup development

Expression of various Wnt signalling components are reported during later eye development [10], [45]–[47], [8] and functionally, the Wnt/β-catenin signaling controls several important processes, such as dorsal retina maintenance or lens cell differentiation [48]–[52]. Furthermore, overexpression of the canonical Wnt signalling and Wnt signalling ablation causes coloboma and microphthalmia [53], [54], suggesting that the Wnt signalling dose is critical for the normal optic cup homeostasis. Interestingly, both, reduction or overexpression of Wnt antagonists, such as *Dkk1* (or *Frizzled5)* results in identical pathologies [19], [55]. These data together suggest the presence of a negative feedback loop between the active Wnt signaling and its limiting via its antagonists in the optic cups. We here support the idea of a direct Wnt/Dkk1 feedback loop, presenting the CNE195 optic cup enhancer as an important locus for this crosstalk. During retina formation, targeting of *Dkk1* by the canonical Wnt signaling might thereby induce a defined Dkk1 level to restrict the Wnt signaling level in the dorsal retina to ensure dorsal retina identity. We found that in addition to Lef1, Msx2 directly targets CNE195, suggesting that Msx2, a downstream effector of Bmp4, favours *Dkk1* expression at the expense of Wnt signaling in the optic cups. In contrast to Msx2, Msx1 interferes poorly with CNE195, suggesting that Msx2 is the major *Dkk1* regulator in this tissue. Together our data strongly suggest that the well defined balance of Wnt signalling activity and the Dkk1 level is critical for proper optic cup formation, supported by the fact that the imbalance of the Dkk1/Wnt doses are associated with several human optic cup pathologies [19], [53]–[55].

### Regulation of *Dkk1* and implications for craniofacial development

The canonical Wnt signalling plays a major role during craniofacial developmental processes, e.g. the species specific outgrowth of the maxillary arches, mandibular bows and the frontonasal process and the fusion and formation of the lips [15], [56]. In line with this, *Dkk1* overexpression leads to reduced outgrowth of the frontonasal mass [15], suggesting a crosstalk between Wnt signalling and *Dkk1* during craniofacial development. Furthermore, Wnt3 and Dkk1 interact in a negative feedback loop during craniofacial development and the interaction between Dkk1 and the canonical Wnt signalling controls the distance between individual hair follicles [57], [16]. We found that the mutagenesis of both conserved Tcf/Lef1 binding sites simultaneously results in a complete loss of reporter expression in all craniofacial expression domains (Fig. 5), suggesting a strong requirement for these sites for a Wnt/*Dkk1* feedback regulation during craniofacial development. However, we also found that the two conserved Tcf/Lef1 binding sites are individually essential for promoting *Dkk1* expression in the whisker hair bud mesenchyme, suggesting an essential role for these CNE114 Tcf/Lef1 sites for the Wnt/Dkk1 crosstalk during hair follicle outgrowth. In addition, Tcf/Lef1 site (no. 2) is individually required for the repression of expression in maxillar mesenchymal domains and activation in the mandibular regions. Since *Dkk1* expression in these domains is present prior to *Lef1* expression (Fig. 1), most likely other factors promote *Dkk1* expression initiation and Lef1 is rather controlling the maintenance. Indeed, several conserved transcription factor binding sites for proteins that are involved in craniofacial development reside within CNE114, such as Dlx1/2/5 and LIM and therefore might recruit Lef1 to its binding sites [34].

Together, we here demonstrate that *Dkk1* reporter expression correlates with the binding of Lef1 and Msx1/2 transcription factors to their conserved binding sites *in vivo*, however, its functional relevance needs to be tested in future analyses.

## Supporting Information

S1 Fig **LiCl treatment of MEFs stimulates** ***Dkk1*** **expression and Lef1 binds to Tcf/Lef1 sites localized within the** ***Dkk1*** **promoter.** Quantitative changes of *Dkk1* expression in mouse embryonic fibroblasts (MEFs) without LiCl treatment and after LiCl treatment as indicated. *Dkk1* expression is significantly enhanced after a LiCl treatment of 50 mM or higher. (B–E) ChIP assays on E12.5 crosslinked craniofacial (B+C) and optic cup (D+E) DNA using Lef1 specific antibodies. *Dkk1* promoter specific (B+D) and *Dkk1* exon 4 specific (C+E) primers were used for amplification. An input DNA fraction and IgG antibodies, directed against RNA polymerase II controls are indicated. Lef1 specifically interacts with the non-conserved Tcf/Lef1 binding sites located within the *Dkk1* promotor in both, the craniofacial domains (B) and the optic cups (D). Lef1 does not interfere with the conserved exonic Lef1 binding site in both tissues (C+E). Representative results of at least three independently performed ChIP assays are presented in this figure.
(TIF)

S2 Fig **Reporter gene expression in transgenic embryos after pronuclear injection of modified CNE195 constructs.** (A–L) X-gal staining of E12.5 transient transgenic embryos after pronuclear injection of CNE195Del1 (A, E, I), CNE195MutTcf/Lef1 (B, F, J), CNE195Del2 (C, G, K), CNE195MutMsx (D, H, L) reporter constructs. A–D represent whole mount side views, E–H show dorsal views to the brain and I-L show whole mount staining in the dissected metanephros.
(TIF)

S3 Fig **Reporter gene expression in transgenic embryos after pronuclear injection of modified CNE114 constructs.** (A–I) X-gal stainings of E12.5 transgenic embryos after pronuclear injection of CNE114 Del (A+B), CNE114 Mut Tcf/Lef1 (no. 2) (C+D) and CNE114 Mut Tcf/Lef1 (no. 1+2) (E–I). A, C, E represent whole mount side views, B, D, F show dorsal views to the brain. (G+I) transverse sections of X-gal stained embryos after injection of the CNE114 Mut Tcf/Lef1 construct. Arrows point to the reporter expression in the otic vesicles (E+G) and the midbrain (F, H, I).
(TIF)
