# Supplementary figures and images for: Differential Binding of Lef1 and Msx1/2 Transcription Factors to Dkk1 CNEs Correlates with Reporter Gene Expression In Vivo
Source: PLoS One. 2014 Dec 29;9(12):e115442. doi: 10.1371/journal.pone.0115442 (PMC4278905; doi:10.1371/journal.pone.0115442)

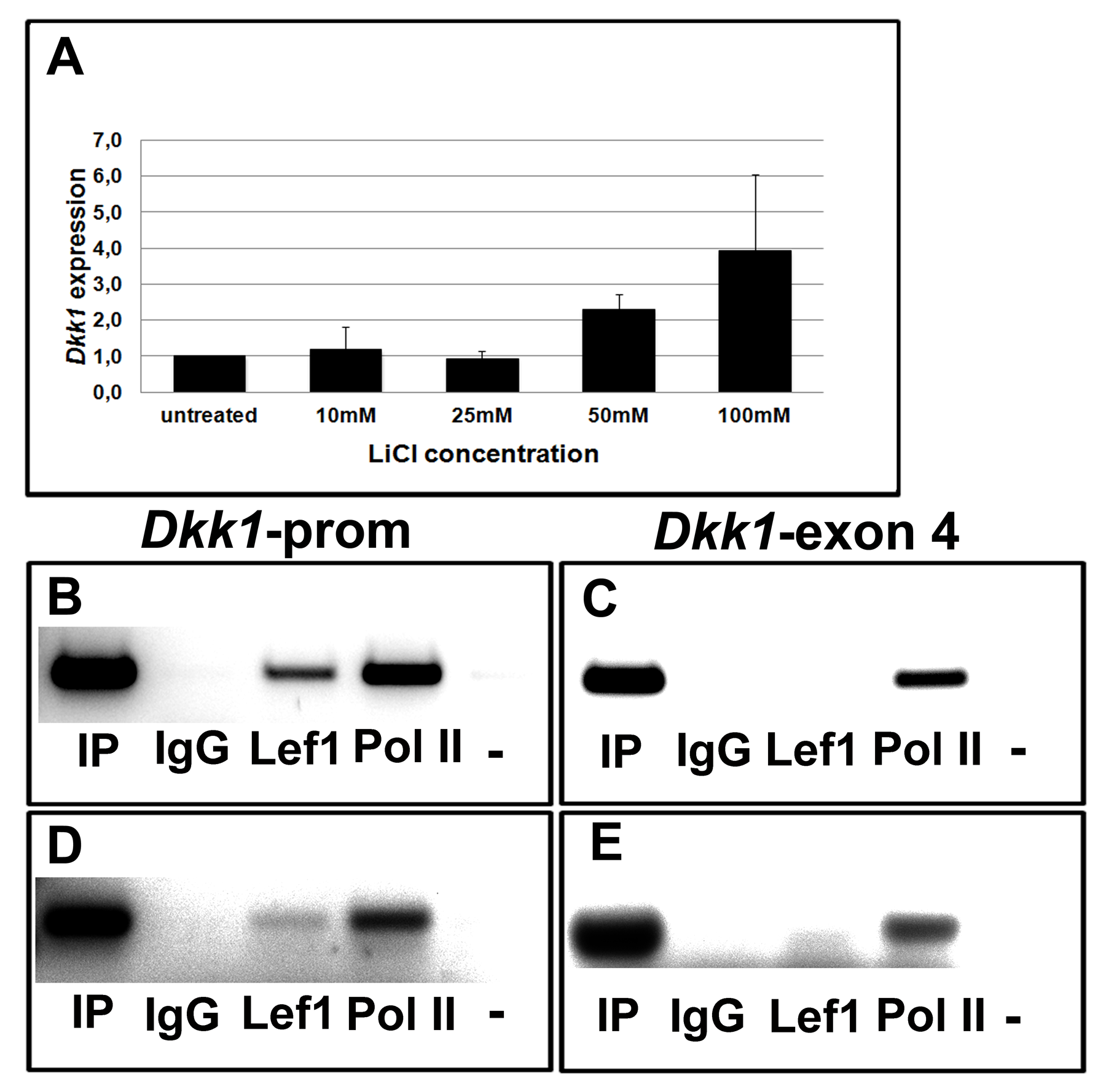

Supplement: S1 Fig — LiCl treatment of MEFs stimulates Dkk1 expression and Lef1 binds to Tcf/Lef1 sites localized within the Dkk1 promoter. Quantitative changes of Dkk1 expression in mouse embryonic fibroblasts (MEFs) without LiCl treatment and after LiCl treatment as indicated. Dkk1 expression is significantly enhanced after a LiCl treatment of 50 mM or higher. (B–E) ChIP assays on E12.5 crosslinked craniofacial (B+C) and optic cup (D+E) DNA using Lef1 specific antibodies. Dkk1 promoter specific (B+D) and Dkk1 exon 4 specific (C+E) primers were used for amplification. An input DNA fraction and IgG antibodies, directed against RNA polymerase II controls are indicated. Lef1 specifically interacts with the non-conserved Tcf/Lef1 binding sites located within the Dkk1 promotor in both, the craniofacial domains (B) and the optic cups (D). Lef1 does not interfere with the conserved exonic Lef1 binding site in both tissues (C+E). Representative results of at least three independently performed ChIP assays are presented in this figure. (TIF) [file pone.0115442.s001.tif]

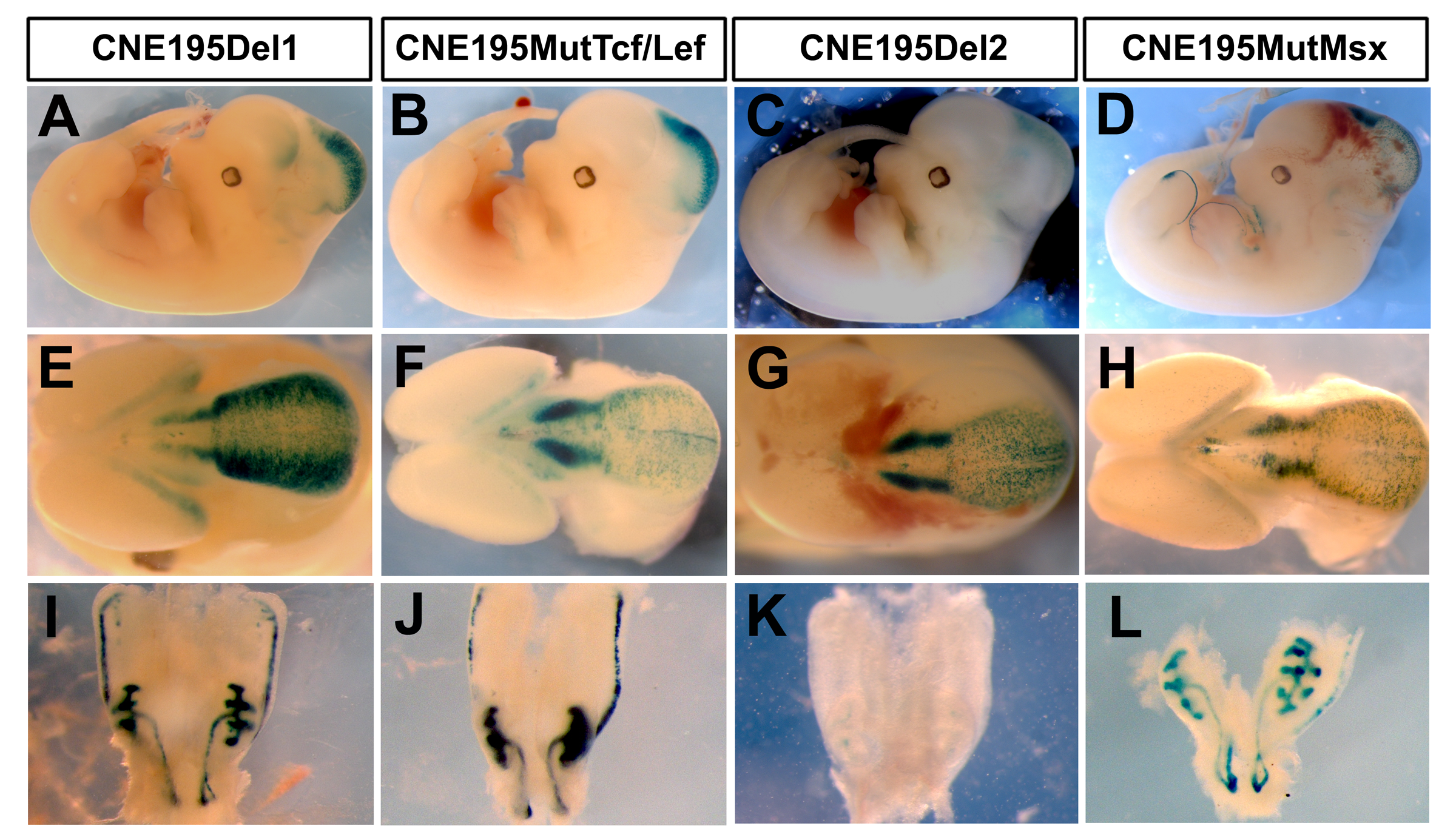

Supplement: S2 Fig — Reporter gene expression in transgenic embryos after pronuclear injection of modified CNE195 constructs. (A–L) X-gal staining of E12.5 transient transgenic embryos after pronuclear injection of CNE195Del1 (A, E, I), CNE195MutTcf/Lef1 (B, F, J), CNE195Del2 (C, G, K), CNE195MutMsx (D, H, L) reporter constructs. A–D represent whole mount side views, E–H show dorsal views to the brain and I-L show whole mount staining in the dissected metanephros. (TIF) [file pone.0115442.s002.tif]

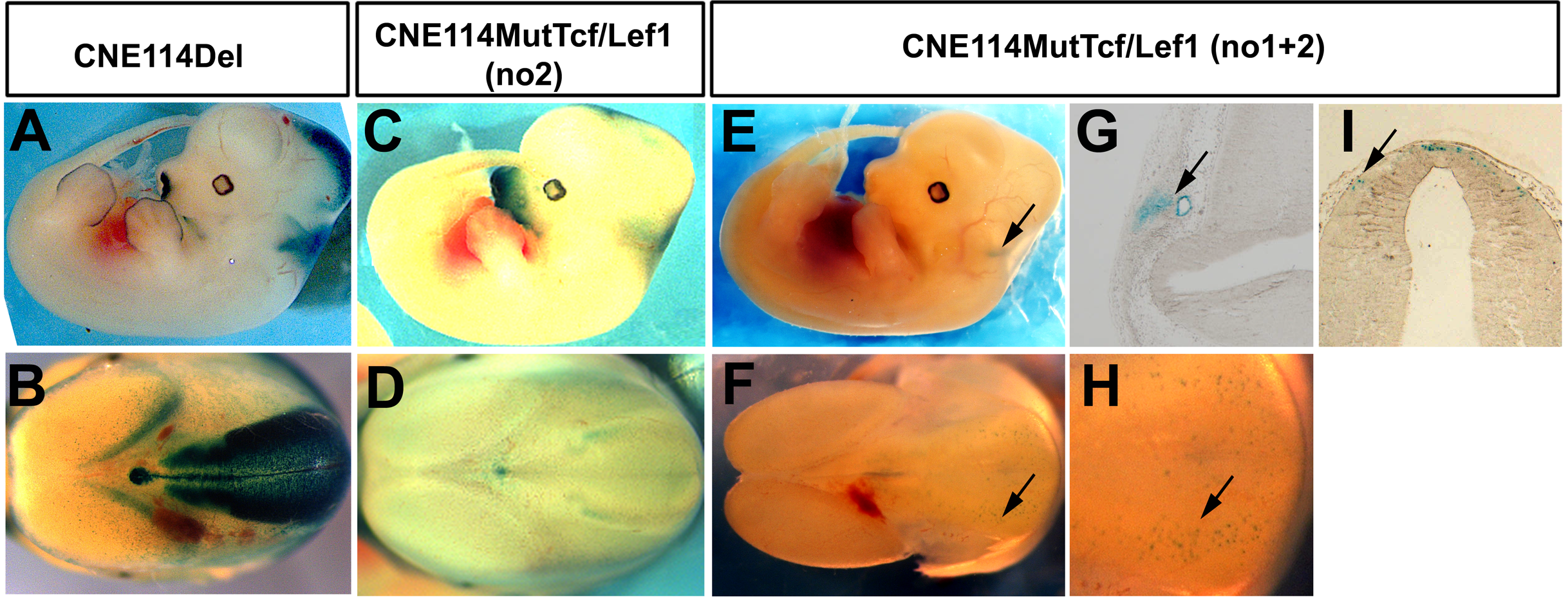

Supplement: S3 Fig — Reporter gene expression in transgenic embryos after pronuclear injection of modified CNE114 constructs. (A–I) X-gal stainings of E12.5 transgenic embryos after pronuclear injection of CNE114 Del (A+B), CNE114 Mut Tcf/Lef1 (no. 2) (C+D) and CNE114 Mut Tcf/Lef1 (no. 1+2) (E–I). A, C, E represent whole mount side views, B, D, F show dorsal views to the brain. (G+I) transverse sections of X-gal stained embryos after injection of the CNE114 Mut Tcf/Lef1 construct. Arrows point to the reporter expression in the otic vesicles (E+G) and the midbrain (F, H, I). (TIF) [file pone.0115442.s003.tif]
